# Intragenic *Agrobacterium*-mediated gene transfer mimics micro-translocations without foreign DNA

**DOI:** 10.1007/s00425-024-04329-x

**Published:** 2024-02-06

**Authors:** Philippa J. Barrell, Julie M. Latimer, Timothy R. Millar, Jeanne M. E. Jacobs, Anthony J. Conner

**Affiliations:** 1 New Zealand Institute for Plant and Food Research Ltd, Private Bag 4704, Christchurch, New Zealand; 2 AgResearch Ltd, Private Bag 4749, Christchurch, New Zealand

**Keywords:** Acetohydroxyacid synthase, Herbicide resistance, Intragenic vectors, Micro-translocations, *Nicotiana tabacum*

## Abstract

**Main conclusion:**

*Agrobacterium*-mediated transformation of *Nicotiana tabacum*, using an intragenic T-DNA region derived entirely from the *N. tabacum* genome, results in the equivalence of micro-translocations within genomes.

**Abstract:**

Intragenic *Agrobacterium*-mediated gene transfer was achieved in *Nicotiana tabacum* using a T-DNA composed entirely of *N. tabacum* DNA, including T-DNA borders and the *acetohydroxyacid synthase* gene conferring resistance to sulfonylurea herbicides. Genomic analysis of a resulting plant, with single locus inheritance of herbicide resistance, identified a single insertion of the intragenic T-DNA on chromosome 5. The insertion event was composed of three *N. tabacum* DNA fragments from other chromosomes, as assembled on the T-DNA vector. This validates that intragenic transformation of plants can mimic micro-translocations within genomes, with the absence of foreign DNA.

**Supplementary Information:**

The online version contains supplementary material available at 10.1007/s00425-024-04329-x.

## Introduction

The intragenic vector concept involves the identification of functional vector elements within plant genomes and their assembly into vectors for gene transfer to plants (Conner et al. 2007). For the transfer of genes within the gene pools available to plant breeders, this allows genetic modification without the introduction of foreign DNA (Barrell et al. 2010). It thereby addresses many of the ethical and/or public concerns raised in the genetic engineering debate (Nielsen 2003; Dixon 2005; Myskja 2006). Although intragenic vectors have been developed for numerous plant species and demonstrated to be functional for gene transfer (Chaurasia and Kole 2022), the promise of gene transfer without the introduction of foreign DNA remains to be experimentally validated by genomic analysis of the resulting transformed plants.

Using *Nicotiana tabacum* (tobacco) as a model system, we report the construction of an intragenic T-DNA region, including functional T-DNA borders and a gene conferring herbicide resistance, and demonstrate the recovery of herbicide-resistant *N. tabacum* plants. Thorough genomic analysis of a plant resulting from *Agrobacterium*-mediated gene transfer confirmed the absence of non-*N. tabacum* DNA and the transfer of three conjoined DNA fragments into a single site on a different chromosome. This example validates that intragenic transformation of plants can be achieved with the absence of foreign DNA and can mimic micro-translocations within genomes.

## Materials and methods

### Bioinformatic searches for border-like sequences in *Nicotiana tabacum*

Using a common 24 bp T-DNA consensus sequence: 5′GRCAGGATATATNNNNNKGTMAWS3′ (where R = G or A, K = T or G, S = G or C, M = C or A, W = A or T and N = any nucleotide) (Conner et al. 2007; Barrell et al. 2010), public EST databases were searched for the two motifs, 5′GRCAGGATATAT3′ and 5′KGTMAWS3′, that flank the five-nucleotide variable region. All possible nucleotide configurations of these two motifs were used to search plant EST databases using BLAST (Altschul et al. 1997), while limiting the results to *N. tabacum*. The search parameters included expect values of 10,000 for the larger motif or 20,000 for the shorter motif and default settings.

### Intragenic vector construction

Following the identification of *N. tabacum* ESTs that could be ligated to form a T-DNA-like sequence, a *N. tabacum AHAS* (*acetohydroxyacid synthase*) gene (also known as *acetolactate synthase* or *ALS*) conferring resistance to chlorsulfuron (Lee et al. 1988) was identified for inclusion within the *N. tabacum*-derived T-DNA. The *AHAS* (*SuRB*) gene was selected based on Genbank accession FJ649655 (Townsend et al. 2009) which currently maps to unplaced scaffold NW_015931609.1 of assembly Ntab-TN90 (https://www.ncbi.nlm.nih.gov/gene/107799742/#reference-sequences, downloaded 14 November 2023). A region including 1 kb of the 5′ promoter region and 300 bp of the 3′ terminator was assembled in silico into the *N. tabacum*-derived T-DNA. Two point mutations identified in *N. tabacum* to confer resistance to sulfonylurea herbicides (Lee et al. 1988) were incorporated into the design. The entire 3733 bp sequence (Supplementary Fig. S1) was flanked by *Sal*I sites, and a synthesized DNA fragment, cloned into the plasmid pUC57, was obtained from Genscript Corporation (Piscataway, NJ, USA). Using standard protocols for DNA manipulations (Sambrook et al. 2001) and following manufacturers’ recommendations where appropriate, the construct containing the *N. tabacum*-derived T-DNA region including the *AHAS* gene (Supplementary Fig. S1) was ligated to the 7998 bp *Sal*I backbone of the binary vector pART27 (Gleave 1992). The resulting *N. tabacum* intragenic vector, pTOBIVAHAS (Supplementary Fig. S2), was transferred to *Agrobacterium tumefaciens* strain EH105 (Hood et al. 1993) using the freeze–thaw method (Höfgen and Willmitzer 1988). Alignments and the vector map were generated using Geneious software v10.0.9 (Kearse et al. 2012).

### *N. tabacum* transformation

*A. tumefaciens* EHA105 harbouring pTOBIVAHAS was cultured at 28 °C on an orbital shaker in LB broth supplemented with 300 mg L^−1^ spectinomycin. *N. tabacum* (cv. Petit Havana SR1) transformation essentially followed the standard leaf-disc transformation protocol (Horsch et al. 1985), except 20 μg L^−1^ chlorsulfuron was used to select for transformed shoots. Regenerated shoots were subcultured onto MS salts (Murashige and Skoog 1962) with 30 g L^−1^ sucrose, 0.8% agar (Standard Grade, New Zealand Manuka Group), 200 mg L^−1^ Timentin and 7.5 μg L^−1^ chlorsulfuron. All cultures were grown under cool, white fluorescent lights (30 μmol m^−2^ s^−1^; 16-h photoperiod) with temperature controlled at a constant 22 °C.

### Inheritance of chlorsulfuron resistance

Plants were transferred from tissue culture into seed raising mix (Yates, Auckland, New Zealand), and grown in a growth room under cool, white fluorescent light (30 μmol m^−2^ s^−1^; 16-h photoperiod), with temperature controlled at 23 °C (day) and 18 °C (night). Intragenic plants were self-pollinated, and reciprocal backcrosses with wild-type plants were performed. Flowers used as ovule parents were emasculated with fine forceps prior to anther dehiscence and immediately pollinated by brushing dehiscent anthers from other flowers against receptive stigmas. Seed capsules were harvested when their apices had started to turn brown, but well before capsule dehiscence, approximately 4–5 weeks after pollination. Seeds were surface sterilized with 96% ethanol for 30 s, followed by 0.2% hypochlorite for 5 min, and then rinsed three times in sterile water. Seeds were plated onto quarter strength MS salts plus 0.8% agar and 20 μg L^−1^ chlorsulfuron. *N. tabacum* seedlings were assessed for chlorsulfuron resistance seven days after sowing. Resistant seedlings had upright large cotyledons with first true leaf appearing and roots penetrating the medium with prominent root hairs, while susceptible seedlings were collapsed onto the surface of the medium with small cotyledons and a short, curled root.

### Genomic analysis

Genomic DNA was isolated from the *N. tabacum* plants using a NucleoSpin® Plant II (Macherey–Nagel, Düren, Germany) kit according to the manufacturer’s instructions. To determine whether plants contained the plant-derived T-DNA, primers were designed to amplify across the junction between the left border EST and the start of the *AHAS* promoter sequence. These were TobF1 and TobR1 (Supplementary Table S1) and had an expected amplification product of 422 bp. To determine whether vector backbone had incorporated into the genome, the primers BBF1 and BBR1 were used (Supplementary Table S1) that produce an expected amplification product of 210 bp. PCRs were performed in 15 μL volumes, with primers at a final concentration of 0.5 μM, magnesium chloride at 2.0 mM, 1 × PCR buffer, 1 × dNTPs (Thermo Scientific, Waltham, MA, USA) and 0.5 units of Taq polymerase. PCR amplification conditions comprised 95 °C for 2 min, then 39 cycles of 95 °C for 30 s, 58 °C for 30 s, followed by 1.5 min at 72 °C. PCR products were electrophoresed in 1.5% agarose in 1 × TAE buffer and visualized after staining with ethidium bromide.

Genomic DNA from one chlorsulfuron-resistant seedling from each regenerated line was sent to Novogene (Hong Kong) for whole genome sequencing. Novogene constructed whole genome shotgun libraries (350 bp short insert) using a DNA Library Prep Kit (NEBNext®, NEB, Ipswich, MA, USA). Read pairs of 150 bp were generated on a NovaSeq 6000. Adapter sequences were removed by the sequence provider. Wild-type *N. tabacum* DNA paired-end sequences from three genotypes were downloaded from the Sequence Read Archive (https://www.ncbi.nlm.nih.gov/sra, downloaded February 2022). The samples were *N. tabacum* TN90 (SRR955756), *N. tabacum* K326 (SRR955771) and *N. tabacum* Basma Xanthi (SRR955782).

The short read whole genome sequences from two intragenic and the three wild-type samples were analysed using a programme originally designed to map transposable elements (TEs), TE Fingerprint (https://github.com/PlantandFoodResearch/TEFingerprint). TE Fingerprint is a command line tool for producing TE-based fingerprints from paired-end reads that allows the easy comparison of different samples. The outputs from TE Fingerprint are plain text format files (GFF3 and tabular format).

To determine the intragenic T-DNA insertion sites of the intragenic plants, TE Fingerprint was used to map the paired-end reads to sections of the vector used to transform *N. tabacum* (pTOBIVAHAS, Supplementary Fig. S2) instead of mapping paired-end reads to a library of TEs. The fasta files of vector sequence are shown in Supplementary Fig. S3. TE Fingerprint first isolated paired-end reads where one end mapped to the intragenic vector, then filtered out any paired-end reads in which neither or both ends mapped to the vector sequence. In a second step, the other end of the paired-end reads was mapped to the *N. tabacum* reference genome (Edwards et al. 2017). BAM files and outputs from TE Fingerprint were then visualized using Integrative Genomics Viewer (V2.15.2) (Robinson et al. 2011).

### DNA sequence analysis across integration sites of intragenic plants

Primers were designed based on the chromosomal location detected using TE Fingerprint. These were TobChr5_10058233 and TobChr5_10058662 (Supplementary Table S1). The numerical part of the primer name indicates where the primer binds on *N. tabacum* chromosome 5 of the reference genome (Edwards et al. 2017). The PCR amplification conditions were the same as described above and products were excised from the agarose gels and purified using a Nucleospin Gel and PCR Clean-up kit (Macherey–Nagel). Approximately 10 ng of purified PCR product and 4 pM of primer per reaction was sent to the Massey Genome Service (Massey University, Palmerston North, New Zealand) for Sanger sequencing. Sequencing reactions were analysed using Geneious software v10.0.9 (Kearse et al. 2012).

## Results

Four *N. tabacum* sequences were identified for assembly of T-DNA border-like sequences. Adjoining part of the EST sequences FS404630 and FS404609 provided a left T-DNA border-like sequence, and adjoining part of the ESTs FS378669 and FS390135 provided a right T-DNA border-like sequence. Insertion between these two T-DNA borders of a *N. tabacum AHAS* (*acetohydroxyacid synthase*) gene conferring resistance to chlorsulfuron resulted in a *N. tabacum* intragenic T-DNA (Supplementary Fig. S1). A map of the entire binary vector is illustrated in Supplementary Figure S2. Following a single co-cultivation experiment with *A. tumefaciens* harbouring pTOBIVAHAS, approximately 60 explants were distributed across five tissue culture plates. From these explants 11 transformed *N. tabacum* plants with high growth on chlorsulfuron-containing medium were recovered. PCR results indicated that four plants were transformed with the intragenic T-DNA and likely to be vector backbone free, of which two (021401-1 and 021401-4) survived to flowering. Inheritance of chlorsulfuron resistance in both plants was not significantly different from expected ratios for a single dominant locus following self-pollination or backcrossing with wild-type plants (Table 1).

**Table 1:** Inheritance of chlorsulfuron resistance in the intragenic *Nicotiana tabacum* plants

Ovule parent	Pollen parent	Number of resistant progeny	Number of susceptible progeny	Expected ratio of resistant: sensitive progeny	Chi square value	*P*-value for 1 df
021401-1	021401-1	54	15	3:1	0.39	0.53
021401-4	021401-4	67	18	3:1	0.66	0.41
021401-4	021401-4	74	23	3:1	0.08	0.76
Wild-type	021401-1	28	26	1:1	0.07	0.78
Wild-type	021401-1	60	46	1:1	1.84	0.17
Wild-type	021401-4	40	41	1:1	0.01	0.91
021401-1	Wild-type	39	37	1:1	0.05	0.81
021401-4	Wild-type	42	37	1:1	0.31	0.57
021401-4	Wild-type	58	52	1:1	0.32	0.56
Wild-type	Wild-type	0	188	–	–	
Wild-type	Wild-type	0	59	–	–	

Each row represents data from independent pollination events (single capsules) of intragenic and wild-type plants

**Fig. 1:**
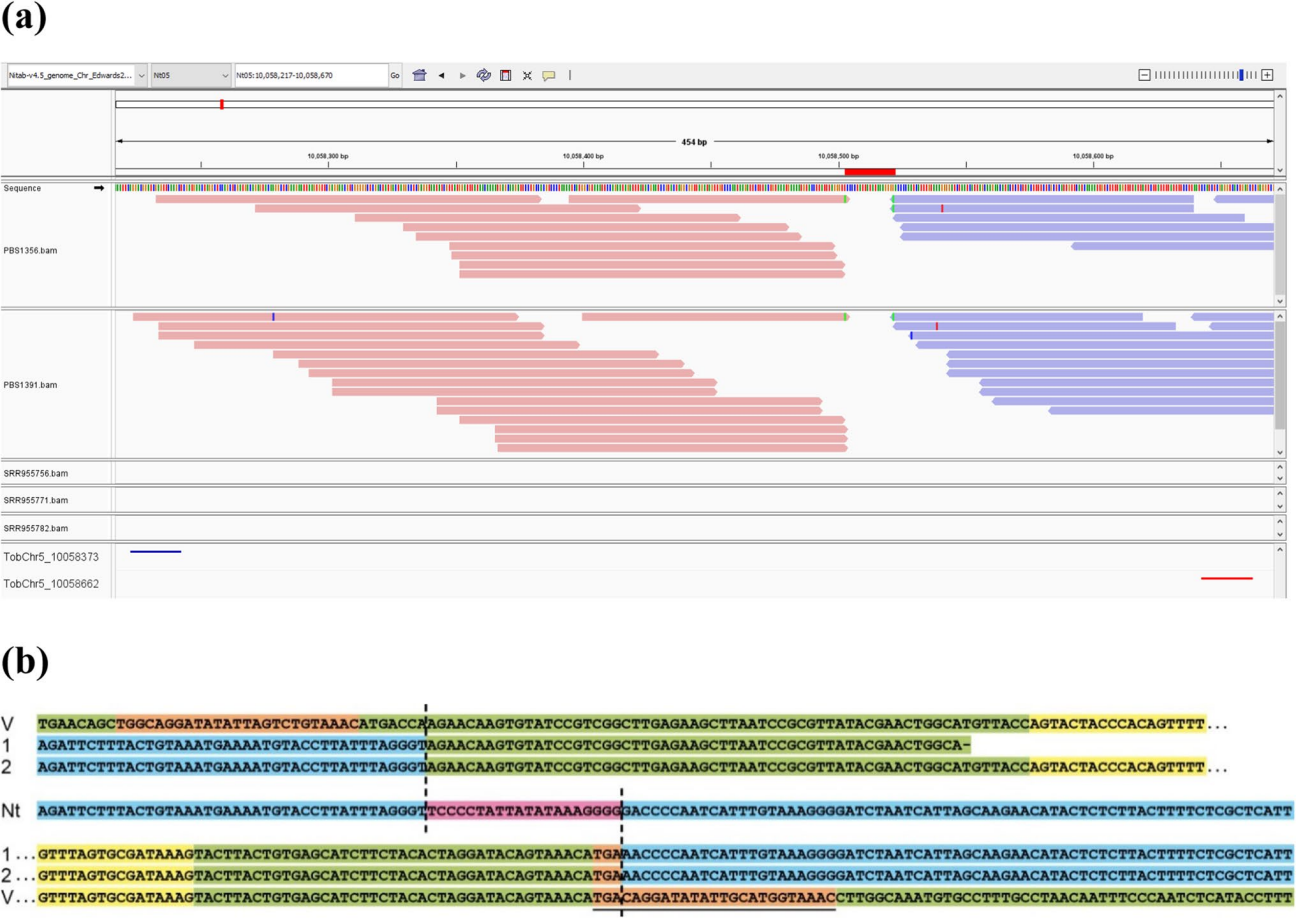
Analysis of the intragenic T-DNA insertion in the *Nicotiana tabacum* genome. **a** Output from TE Fingerprint visualized with Integrative Genomics Viewer (Robinson et al 2011). The two intragenic plant samples PBS1356 and PBS1391 have forward reads mapped in pink and reverse reads mapped in blue. The red bar indicates the 20 bp deletion at the intragenic insertion site. As expected, TE Fingerprint did not identify paired-end sequences on chromosome 5 using the wild-type libraries SRR955771 (K326), SRR955782 (Basma Xanthi) and SRR955756 (cultivar TN90). PCR primer binding sites for confirmation of the intragenic insertion site are indicated at the bottom of the figure. **b** Alignment of sequenced PCR products from chromosome 5 of two *N. tabacum* intragenic plants (1 and 2) with the intragenic vector (V) and chromosome 5, as shown in **a**, from the *N. tabacum* reference genome (Nt) (Edwards et al 2017). PCR amplifications contained one primer specific to chromosome 5 and one specific to the intragenic vector in the region of each border (Supplementary Table S2). Chromosome 5 is highlighted in blue, the integration/deletion site in pink, *N. tabacum*-derived vector in green, *N. tabacum*-derived T-DNA borders in orange (right border underlined) and *N. tabacum* AHAS sequence in yellow

DNA sequence results from single chlorsulfuron-resistant seedlings of PBS 1356 (selfed-seed from plant 021401-1) and PBS 1391 (selfed-seed of plant 021401-4) resulted in 120 Gb raw data per sample. Using TE Fingerprint (https://github.com/PlantandFoodResearch/TEFingerprint), the paired-end sequence reads were mapped to the intragenic vector sequence. No read pairs mapped to the bacterial backbone sequence, confirming the PCR results that the two original plants (021401-1 and 021401-4) were vector backbone free. When one read of the paired-end reads mapped to the intragenic vector sequence, the other read was then mapped to the *N. tabacum* genome (Edwards et al. 2017). This found the same potential single insertion event located within bases 10,058,500–10,058,530 on chromosome 5 of the *N. tabacum* genome for both intragenic lines 021401-1 and 021401-4, indicating they likely arose as clones from a single transformation event (Fig. 1a).

Sequence analysis of PCR-amplified products from chromosome 5 of the transformed intragenic plants (Fig. 1b) revealed that only the first three bases of the right border integrated into the *N. tabacum* genome and that the T-DNA insert had truncated 7 bp prior to the left border. Further sequence analysis revealed a small deletion of 20 bp at the site of integration (Fig. 1b). The insertion site of the *N. tabacum*-derived T-DNA on chromosome 5 involves an intergenic region between an uncharacterized ribosomal protein S2 gene and a Zinc finger gene. Our sequence analysis also revealed that the intragenic T-DNA is present as a single intact copy and that no DNA sequence of bacterial origin was incorporated in the intragenic plants.

## Discussion

To construct an intragenic T-DNA region, only short DNA fragments need to be adjoined for assembly of functional T-DNA borders. Such short sequences are known to exist in many species (Conner et al. 2007; Barrell et al. 2010). The fragments from the *N. tabacum* genome used to gain this T-DNA functionality involved much longer sequences (Supplementary Figs S1, S3) that could only be considered as being of *N. tabacum* origin. The assembled intragenic vector (Supplementary Fig. S2) was highly effective for *Agrobacterium*-mediated transformation of *N. tabacum* and allowed the recovery of at least one transformed shoot per five explants. This approach enables gene transfer without the introduction of foreign DNA and is widely considered as being more acceptable than other forms of genetic modification (Dayé et al. 2023).

Plant transformation is unpredictable in terms of gene integration and expression (Conner and Christey 1994). The nature of integration can occur with complete, truncated or rearranged insertions at single or multiple genome sites. The site of insertion can influence position effects associated with the magnitude, specificity and stability of expression of the transferred gene. Off-types can result from mutational events induced by gene insertion into genomes or somaclonal variation during the tissue culture phase of transformation. Consequently, the use of gene transformation in crop improvement is well recognized as requiring large numbers of initially transformed plants to find one event that meets the requirements for further development and release (Conner and Christey 1994). In this context intragenic transformation is no different to transgenic transformation (Conner et al. 2007), so we simulated this approach in this study. We initially imposed stringent selection to identify 11 transformed *N. tabacum* lines with high phenotypic resistance to chlorsulfuron. Seven of these were eliminated due to the integration of vector backbone sequences beyond the intragenic T-DNA. Two of the remaining lines were poor growing off-types. The remaining two lines exhibited the single locus inheritance of chlorsulfuron resistance among the progeny of these plants (Table 1). This was consistent with the genomic analysis that identified a single insertion site of the intragenic T-DNA in the *N. tabacum* genome (Fig. 1). The identical genomic analysis for these two lines established that they arose as clones from a single transformation event.

Sequence analysis of PCR-amplified products from across the integration site of the *N. tabacum*-derived T-DNA (Fig. 1b) confirmed that the plant-derived border sequences behaved in an equivalent manner to *Agrobacterium*-derived T-DNA border sequences. In the intragenic plants only the first three bases of the right border integrated into the plant genome and the T-DNA truncated 7 bp prior to the left border. This is consistent with *Agrobacterium*-mediated gene transfer where a single strand nick between the third and fourth nucleotides of the right border initiates T-strand synthesis resulting in only three nucleotides of the right border usually being transferred into plant genomes, with truncation occurring on or about the left border (Gheysen et al. 1998; Gelvin 2003). The sequence analysis also revealed a small 20 bp deletion at the integration site (Fig. 1b). Such small deletions are common at *Agrobacterium* T-DNA integration sites (van Kregten et al. 2016; Gelvin 2021). Analysis of thousands of T-DNA insertion sites revealed deletions of up to 100 bp were observed in 86% of insertions, with a median of 19 bp (Kleinboelting et al. 2015).

**Fig. 2:**
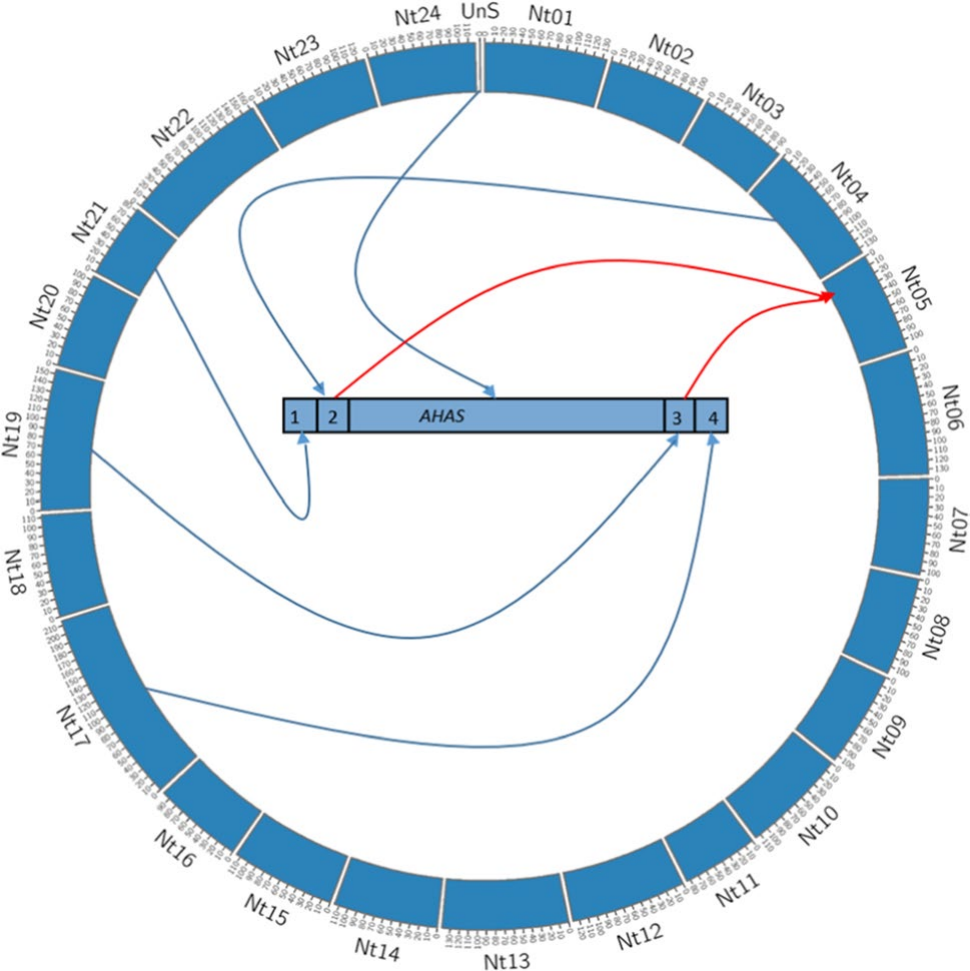
*Nicotiana tabacum* genome translocation map. Circos map (Krzywinski et al. 2009) of *N. tabacum* genome fragments translocated in the intragenic lines. The *N. tabacum* chromosomes are numbered, with UnS representing the unplaced scaffold NW_015931609 which contains the *N. tabacum AHAS* gene. The five blue arrows indicate the chromosome fragments assembled into the *N. tabacum* intragenic T-DNA (1, 2, AHAS, 3, 4), whereas the red arrows indicate the intragenic T-DNA region inserted into chromosome 5 by *Agrobacterium*-mediated gene transfer

The generation of intragenic plants described in this study is synonymous with micro-translocations occurring within genomes (Conner et al. 2007; Barrell et al. 2010). To demonstrate this, the chromosomal locations of the DNA fragments that were conjoined to assemble the intragenic T-DNA and their subsequent integration were used to generate a Circos plot (Krzywinski et al. 2009) (Fig. 2). This illustrates the construction of the intragenic T-DNA from DNA fragments originating from five distinct regions of the *N. tabacum* genome, followed by the *Agrobacterium*-mediated transfer of 3495 bp from three of these conjoined fragments into a site on chromosome 5. This micro-translocation involved 65 bp from FS404609 of chromosome 4, 3386 bp of the *AHAS* gene from an, as yet, unplaced scaffold and 44 bp from FS378669 of chromosome 19.

Plant-derived T-DNA sequences necessary to construct binary vectors for *Agrobacterium*-mediated transformation have been identified in many species (Baldwin et al. 2006). These have proved highly effective when used in plant transformation (Conner et al. 2007; Barrell et al. 2010), which is expected given that the T-DNA border sequences are identical to authentic *Agrobacterium* T-DNA borders. Intragenic plant transformation also requires a plant-derived selectable marker gene, with a sulfonylurea-resistant form of the *N. tabacum* acetohydroxyacid synthase gene used in this study. Similar sulfonylurea-resistant forms of this gene are available within the gene pool of many crop species (Murphy and Tranel 2019; Tranel et al. 2023). If necessary, they can be derived via seed mutagenesis (Conner et al. 1994) or somatic cell selection (Barrell et al. 2017). This provides an opportunity for intragenic vectors to be developed and applied in diverse crop species for the rapid transfer of genes, including those conferring disease resistance and quality traits, directly into elite cultivars.

Intragenic vectors allow the development of genetically transformed plants using only DNA from the gene pool available to plant breeders (Barrell et al. 2010). For well over a decade, intragenic gene transfer has promised a ‘clean’ DNA delivery system. This study has finally validated that intragenic transformation can mimic micro-translocations within genomes, with the absence of foreign DNA in the derived plants. The resulting plants are equivalent to micro-translocations in crop genomes that may arise naturally or through radiation-induced mutation. Biologically, intragenic vector-derived plants are not ‘transgenic’, although they are derived using the tools of molecular biology and plant transformation. The use of intragenic vectors raises several important implications for the regulation of transgenic crops. Since the resulting plants contain no foreign DNA, it challenges the legal definition of genetic modification (Myskja 2006) since identical plants could be theoretically derived via more conventional and acceptable approaches (Nielsen 2003). Recent advances in gene editing have resulted in an increasing number of countries exempting plants from genetic modification status when they contain no foreign DNA (Buchholzer and Frommer 2023). Intragenic plants offer the same opportunity and challenge the testing for genetic modification-free plant material because all DNA sequences are already present in existing crops.

### Supplementary Information

Below is the link to the electronic supplementary material.

Supplementary file1 (DOCX 561 KB)

## Data Availability

The authors confirm that all the experimental data are available and accessible via the main text and/or the supplemental information.

## Abbreviations

AHAS: Acetohydroxyacid synthase
EST: Expressed sequence tag
T-DNA: Transfer DNA
TE: Transposable element
