# Supplementary material for: Intragenic Agrobacterium-mediated gene transfer mimics micro-translocations without foreign DNA
Source: Planta. 2024 Feb 6;259(3):61. doi: 10.1007/s00425-024-04329-x (PMC10847175; doi:10.1007/s00425-024-04329-x)
Supplement: Supplementary file 1 — Supplementary file1 (DOCX 561 KB) [file 425_2024_4329_MOESM1_ESM.docx]

**Table S1**

**PCR primer sequences**

| **Primer name** | **Sequence (5' to 3')** | **Binding site** | **Paired with** |
| --- | --- | --- | --- |
| BBF1 | GCCCCGCAGAAGCTCCCATC | Vector backbone | BBR1 |
| BBR1 | CATCGGGGCAGGCACTTGCT | Vector backbone | BBF1 |
| TobF1 | GTGTATCCGTCGGCTTGAGA | Intragenic T-DNA (FS404609) | TobR1 |
| TobR1 | GCCTTTTGGTTGCCTCGTTA | Intragenic T-DNA (*AHAS* promoter) | TobF1 |
| TobRB2 | TGCATCATCTCGCTTTGGTTTTGA | Intragenic T-DNA (*AHAS* terminator) | TobChr5_10058662 |
| TobLB1 | CCCTGGTGAAATTTGCTTTGTCC | Intragenic T-DNA (*AHAS* promoter) | TobChr5_10058233 |
| TobLB3 | TGCCAGTTCGTATAACGCGGATT | Intragenic T-DNA (FS404609) | TobChr5_10058233 |
| TobChr5_10058233 | AGGACGGATCGAGTTCAAGC | *N. tabacum* chromosome 5 | TobLB1 and TobLB3 |
| TobChr5_10058662 | GCTCAGTGACCTCGAGATGG | *N. tabacum* chromosome 5 | TobRB2 |

**Figure S1**

**Map of the *N. tabacum*-derived T-DNA.** ESTs have been conjoined to make T-DNA border sequences flanking an *AHAS* gene conferring sulfonylurea resistance. EST sequences are represented with dark blue bars. The joining of ESTs FS404630 and FS404609 forms the left T-DNA border (upper orange bar), and the joining of FS378669 and FS390135 forms the T-DNA right border (lower orange bar). The *AHAS* sequence is highlighted in yellow. Nucleotide substitutions conferring chlorsulfuron resistance are indicated in pink.


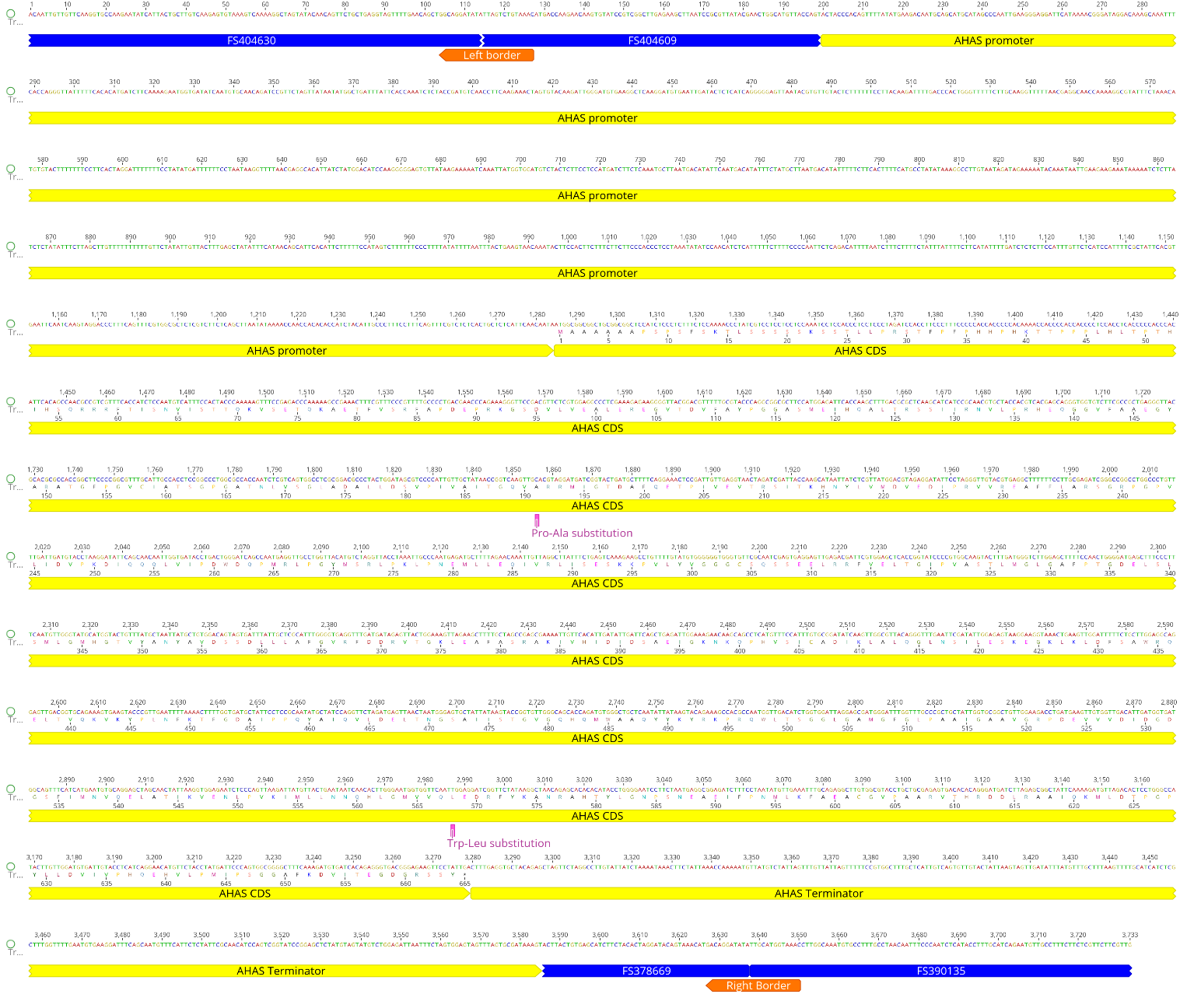


**Figure S2**

**Map of the binary vector with the intragenic *N. tabacum*-derived T-DNA**


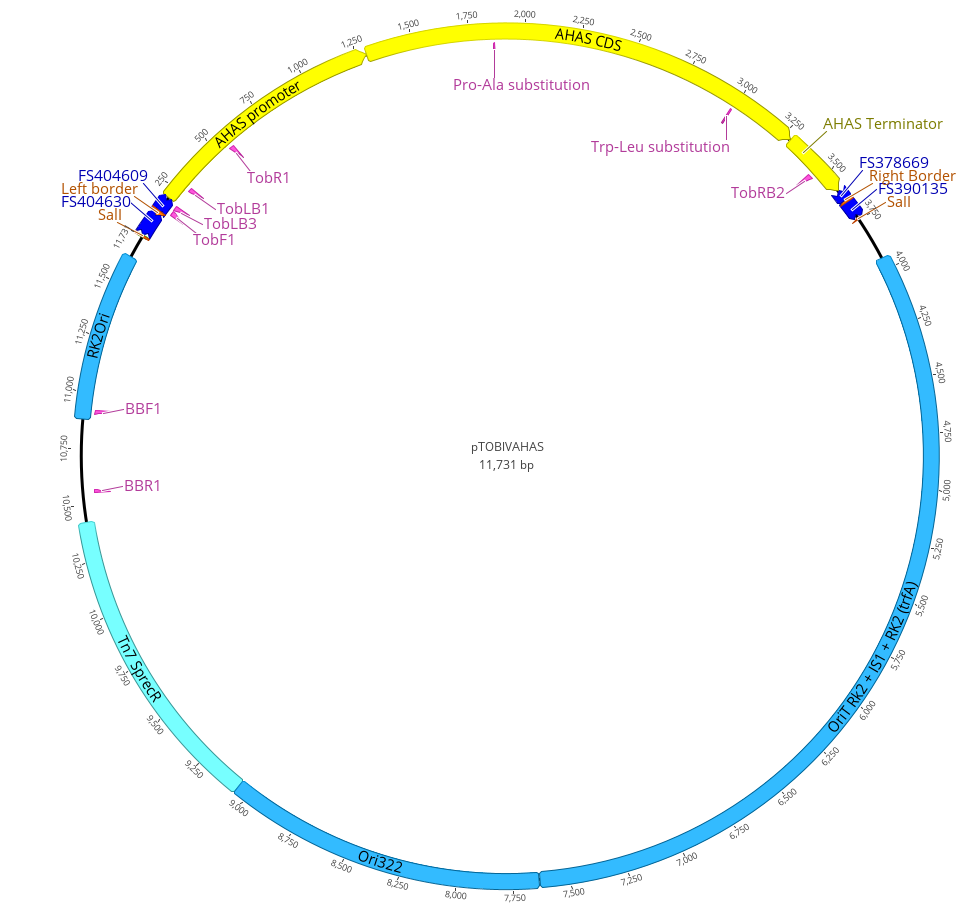


**Figure S3**

**Fasta files of the binary vector pTOBIVAHAS sequence.** These sequences were used with TE Fingerprint to characterize the intragenic *N. tabacum* plants.

>bbrk2ori

GGCGCGGCGTCTATGGCGGCAAAGATGGGAGCTTCTGCGGGGCATTGGTCGTCATAAATGGCCAATTTTCGCTTGCCCTGACAGATAACGGCCTGAAGATCATGTATCTAAGCAACTAGCCTGCTCTCTAATAAAATGTTAGGAGCTTGGCTGCCATTTTTGGGGTGAGGCCGTTCGCGGCCGAGGGGCGCAGCCCCTGGGGGGATGGGAGGCCCGCGTTAGCGGGCCGGGAGGGTTCGAGAAGGGGGGGCACCCCCCTTCGGCGTGCGCGGTCACGCGCCAGGGCGCAGCCCTGGTTAAAAACAAGGTTTATAAATATTGGTTTAAAAGCAGGTTAAAAGACAGGTTAGCGGTGGCCGAAAAACGGGCGGAAACCCTTGCAAATGCTGGATTTTCTGCCTGTGGACAGCCCCTCAAATGTCAATAGGTGCGCCCCTCATCTGTCAGCACTCTGCCCCTCAAGTGTCAAGGATCGCGCCCCTCATCTGTCAGTAGTCGCGCCCCTCAAGTGTCAATACCGCAGGGCACTTATCCCCAGGCTTGTCCACATCATCTGTGGGAAACTCGCGTAAAATCAGGCGTTTTCGCCGATTTGCGAGGCTGGCCAGCTCCACGTCGCCGGCCGAAATCGAGCCTGCCCCTCATCTGTCAACGCCGCGCCGGGTGAGTCGGCCCCTCAAGTGTCAACGTCCGCCCCTCATCTGTCAGTGAGGGCCAAGTTTTCCGCGAGGTATCCACAACGCCGGCGGCCGGCCGCGGTGTCTCGCACACGGCTTCGACGGCGTTTCTGGCGCGTTTGCAGGGCCATAGACGGCCGCCAGCCCAGCGGCGAGGGCAACCAGCCCGGTGAGCGTCGGAAAG

>fs404630

CAATTGTTGTTCAAGGTGCCAAGAATATCATTACTGCTTGTCAAGAGTGTAAAGTCAAAAGGCTAGTATACAACAGTTCTGCTGAGGTAGTTTTGAACAGCTGGCAGGATATA

>fs404609

TTAGTCTGTAAACATGACCAAGAACAAGTGTATCCGTCGGCTTGAGAAGCTTAATCCGCGTTATACGAACTGGCATGTTACCAGT

>surb_promoter_coding_term

ACTACCCACAGTTTTATATGAAGACAATGCAGCATGCATAGCCCAATTGAAGGGAGGATTCATAAAACGGGATAGGACAAAGCAAATTTCACCAGGGTTATTTTTCACACATGATCTTCAAAAGAATGGTGATATCAATGTGCAACAGATCCGTTCTAGTTATAATATGGCTGATTTATTCACCAAATCTCTACCGATGTCAACCTTCAAGAAACTAGTGTACAAGATTGGGATGTGAAGGCTCAAGGATGTGAATTGATACTCTCATCAGGGGGAGTTAATACGTGTTGTACTCTTTTTTCCTTACAAGATTTTGACCCACTGGGTTTTTCTTGCAAGGTTTTTAACGAGGCAACCAAAAGGCGTATTTCTAAACATGTGTACTTTTTTTCCTTCACTAGGATTTTTTTCCTATATGATTTTTTCCTAATAAGGTTTTAACGAGGCACATTATCTATGGACATCCAAGGGGGAGTGTTATAAGAAAAATCAAATTATGGTGGATGTCTACTCTTCCTCCATGATCTTCTCAAATGCTTAATGACATATTCAATGACATATTTCTATGCTTAATGACATATTTTTCTTCACTTTTCATGCCTATATAAAGGCCTTGTAATAGATAGAAAAATACAAATAATTGAAGAAGAAATAAAAATCTCTTATCTCTATATTTCTTAGCTTGTTTTTTTTTTGTTCTATATTGTTACTTTGAGCTATATTTCATAACAGCATTCACATTCTTTTTCCATAGTCTTTTTTCCCTTTTATATTTTAATTTACTGAAGTAACAAATACTTCCACTTCTTTCTTCTTCCCACCCTCCTAAATATATCCAACATCTCATTTTTCTTTTCCCCAATTCTCAGACATTTTAATCTTTCTTTTCTATTTATTTTCTTCATATTTTGATCTCTCTTCCATTTGTTCTCATCCATTTTCGCTATTCACGTGAATTCAATCAAGTAGGACCCTTTCAGTTTCGTGGCGCTCTCGTCTTCTCAGCTTAATATAAAACCAACCACACACCATCTACATTGCCCTTTCCTTTCAGTTTCGTCTCTCACTGCTCTCATTCAACAATAATGGCGGCGGCTGCGGCGGCTCCATCTCCCTCTTTCTCCAAAACCCTATCGTCCTCCTCCTCCAAATCCTCCACCCTCCTCCCTAGATCCACCTTCCCTTTCCCCCACCACCCCCACAAAACCACCCCACCACCCCTCCACCTCACCCCCACCCACATTCACAGCCAACGCCGTCGTTTCACCATCTCCAATGTCATTTCCACTACCCAAAAAGTTTCCGAGACCCAAAAAGCCGAAACTTTCGTTTCCCGTTTTGCCCCTGACGAACCCAGAAAGGGTTCCGACGTTCTCGTGGAGGCCCTCGAAAGAGAAGGGGTTACGGACGTTTTTGCGTACCCAGGCGGCGCTTCCATGGAGATTCACCAAGCTTTGACGCGCTCAAGCATCATCCGCAACGTGCTACCACGTCACGAGCAGGGTGGTGTCTTCGCCGCTGAGGGTTACGCACGCGCCACCGGCTTCCCCGGCGTTTGCATTGCCACCTCCGGCCCTGGCGCCACCAATCTCGTCAGTGGCCTCGCGGACGCCCTACTGGATAGCGTCCCCATTGTTGCTATAACCGGTCAAGTTGCACGTAGGATGATCGGTACTGATGCTTTTCAGGAAACTCCGATTGTTGAGGTAACTAGATCGATTACCAAGCATAATTATCTCGTTATGGACGTAGAGGATATTCCTAGGGTTGTACGTGAGGCTTTTTTCCTTGCGAGATCGGGCCGGCCTGGCCCTGTTTTGATTGATGTACCTAAGGATATTCAGCAACAATTGGTGATACCTGACTGGGATCAGCCAATGAGGTTGCCTGGTTACATGTCTAGGTTACCTAAATTGCCCAATGAGATGCTTTTAGAACAAATTGTTAGGCTTATTTCTGAGTCAAAGAAGCCTGTTTTGTATGTGGGGGGTGGGTGTTCGCAATCGAGTGAGGAGTTGAGACGATTCGTGGAGCTCACCGGTATCCCCGTGGCAAGTACTTTGATGGGTCTTGGAGCTTTTCCAACTGGGGATGAGCTTTCCCTTTCAATGTTGGGTATGCATGGTACTGTTTATGCTAATTATGCTGTGGACAGTAGTGATTTATTGCTCGCATTTGGGGTGAGGTTTGATGATAGAGTTACTGGAAAGTTAGAAGCTTTTGCTAGCCGAGCGAAAATTGTTCACATTGATATTGATTCAGCTGAGATTGGAAAGAACAAGCAGCCTCATGTTTCCATTTGTGCGGATATCAAGTTGGCGTTACAGGGTTTGAATTCGATATTGGAGAGTAAGGAAGGTAAACTGAAGTTGGATTTTTCTGCTTGGAGGCAGGAGTTGACGGTGCAGAAAGTGAAGTACCCGTTGAATTTTAAAACTTTTGGTGATGCTATTCCTCCGCAATATGCTATCCAGGTTCTAGATGAGTTAACTAATGGGAGTGCTATTATAAGTACCGGTGTTGGGCAGCACCAGATGTGGGCTGCTCAATATTATAAGTACAGAAAGCCACGCCAATGGTTGACATCTGGTGGATTAGGAGCGATGGGATTTGGTTTGCCCGCTGCTATTGGTGCGGCTGTTGGAAGACCTGATGAAGTTGTGGTTGACATTGATGGTGATGGCAGTTTCATCATGAATGTGCAGGAGCTAGCAACTATTAAGGTGGAGAATCTCCCAGTTAAGATTATGTTACTGAATAATCAACACTTGGGAATGGTGGTTCAATTGGAGGATCGGTTCTATAAGGCTAACAGAGCACACACATACCTGGGGAATCCTTCTAATGAGGCGGAGATCTTTCCTAATATGTTGAAATTTGCAGAGGCTTGTGGCGTACCTGCTGCGAGAGTGACACACAGGGATGATCTTAGAGCGGCTATTCAAAAGATGTTAGACACTCCTGGGCCATACTTGTTGGATGTGATTGTACCTCATCAGGAACATGTTCTACCTATGATTCCCAGTGGCGGGGCTTTCAAAGATGTGATCACAGAGGGTGACGGGAGAAGTTCCTATTGACTTTGAGGTGCTACAGAGCTAGTTCTAGGCCTTGTATTATCTAAAATAAACTTCTATTAAACCAAAAATGTTATGTCTATTAGTTTGTTATTAGTTTTTCCGTGGCTTTGCTCATTGTCAGTGTTGTACTATTAAGTAGTTGATATTTATGTTTGCTTTAAGTTTTGCATCATCTCGCTTTGGTTTTGAATGTGAAGGATTTCAGCAATGTTTCATTCTCTATTCGCAACATCCAGTCGGTATCCGGAGCTCTATGTAGTATGTCTGGAGATTAATTTCTAGTGGAGTAGTTTAGTGCGATAAAGT

>fs378669

ACTTACTGTGAGCATCTTCTACACTAGGATACAGTAAACATGACAGGATATA

>fs390135

TTGCATGGTAAACCTTGGCAAATGTGCCTTTGCCTAACAATTTCCCAATCTCATACCTTTGCATCAGAATGTTGCCTTTCTTCTCGTTCTTCGTTG

>bboritrk2

TCGACGGATCTTTTCCGCTGCATAACCCTGCTTCGGGGTCATTATAGCGATTTTTTCGGTATATCCATCCTTTTTCGCACGATATACAGGATTTTGCCAAAGGGTTCGTGTAGACTTTCCTTGGTGTATCCAACGGCGTCAGCCGGGCAGGATAGGTGAAGTAGGCCCACCCGCGAGCGGGTGTTCCTTCTTCACTGTCCCTTATTCGCACCTGGCGGTGCTCAACGGGAATCCTGCTCTGCGAGGCTGGCCGGCTACCGCCGGCGTAACAGATGAGGGCAAGCGGATGGCTGATGAAACCAAGCCAACCAGGGGTGATGCTGCCAACTTACTGATTTAGTGTATGATGGTGTTTTTGAGGTGCTCCAGTGGCTTCTGTTTCTATCAGCTGTCCCTCCTGTTCAGCTACTGACGGGGTGGTGCGTAACGGCAAAAGCACCGCCGGACATCAGCGCTATCTCTGCTCTCACTGCCGTAAAACATGGCAACTGCAGTTCACTTACACCGCTTCTCAACCCGGTACGCACCAGAAAATCATTGATATGGCCATGAATGGCGTTGGATGCCGGGCAACAGCCCGCATTATGGGCGTTGGCCTCAACACGATTTTACGTCACTTAAAAAACTCAGGCCGCAGTCGGTAACCTCGCGCATACAGCCGGGCAGTGACGTCATCGTCTGCGCGGAAATGGACGAACAGTGGGGCTATGTCGGGGCTAAATCGCGCCAGCGCTGGCTGTTTTACGCGTATGACAGTCTCCGGAAGACGGTTGTTGCGCACGTATTCGGTGAACGCACTATGGCGACGCTGGGGCGTCTTATGAGCCTGCTGTCACCCTTTGACGTGGTGATATGGATGACGGATGGCTGGCCGCTGTATGAATCCCGCCTGAAGGGAAAGCTGCACGTAATCAGCAAGCGATATACGCAGCGAATTGAGCGGCATAACCTGAATCTGAGGCAGCACCTGGCACGGCTGGGACGGAAGTCGCTGTCGTTCTCAAAATCGGTGGAGCTGCATGACAAAGTCATCGGGCATTATCTGAACATAAAACACTATCAATAAGTTGGAGTCATTACCCAACCAGGAAGGGCAGCCCACCTATCAAGGTGTACTGCCTTCCAGACGAACGAAGAGCGATTGAGGAAAAGGCGGCGGCGGCCGGCATGAGCCTGTCGGCCTACCTGCTGGCCGTCGGCCAGGGCTACAAAATCACGGGCGTCGTGGACTATGAGCACGTCCGCGAGCTGGCCCGCATCAATGGCGACCTGGGCCGCCTGGGCGGCCTGCTGAAACTCTGGCTCACCGACGACCCGCGCACGGCGCGGTTCGGTGATGCCACGATCCTCGCCCTGCTGGCGAAGATCGAAGAGAAGCAGGACGAGCTTGGCAAGGTCATGATGGGCGTGGTCCGCCCGAGGGCAGAGCCATGACTTTTTTAGCCGCTAAAACGGCCGGGGGGTGCGCGTGATTGCCAAGCACGTCCCCATGCGCTCCATCAAGAAGAGCGACTTCGCGGAGCTGGTATTCGTGCAGGGCAAGATTCGGAATACCAAGTACGAGAAGGACGGCCAGACGGTCTACGGGACCGACTTCATTGCCGATAAGGTGGATTATCTGGACACCAAGGCACCAGGCGGGTCAAATCAGGAATAAGGGCACATTGCCCCGGCGTGAGTCGGGGCAATCCCGCAAGGAGGGTGAATGAATCGGACGTTTGACCGGAAGGCATACAGGCAAGAACTGATCGACGCGGGGTTTTCCGCCGAGGATGCCGAAACCATCGCAAGCCGCACCGTCATGCGTGCGCCCCGCGAAACCTTCCAGTCCGTCGGCTCGATGGTCCAGCAAGCTACGGCCAAGATCGAGCGCGACAGCGTGCAACTGGCTCCCCCTGCCCTGCCCGCGCCATCGGCCGCCGTGGAGCGTTCGCGTCGTCTCGAACAGGAGGCGGCAGGTTTGGCGAAGTCGATGACCATCGACACGCGAGGAACTATGACGACCAAGAAGCGAAAAACCGCCGGCGAGGACCTGGCAAAACAGGTCAGCGAGGCCAAGCAGGCCGCGTTGCTGAAACACACGAAGCAGCAGATCAAGGAAATGCAGCTTTCCTTGTTCGATATTGCGCCGTGGCCGGACACGATGCGAGCGATGCCAAACGACACGGCCCGCTCTGCCCTGTTCACCACGCGCAACAAGAAAATCCCGCGCGAGGCGCTGCAAAACAAGGTCATTTTCCACGTCAACAAGGACGTGAAGATCACCTACACCGGCGTCGAGCTGCGGGCCGACGATGACGAACTGGTGTGGCAGCAGGTGTTGGAGTACGCGAAGCGCACCCCTATCGGCGAGCCGATCACCTTCACGTTCTACGAGCTTTGCCAGGACCTGGGCTGGTCGATCAATGGCCGGTATTACACGAAGGCCGAGGAATGCCTGTCGCGCCTACAGGCGACGGCGATGGGCTTCACGTCCGACCGCGTTGGGCACCTGGAATCGGTGTCGCTGCTGCACCGCTTCCGCGTCCTGGACCGTGGCAAGAAAACGTCCCGTTGCCAGGTCCTGATCGACGAGGAAATCGTCGTGCTGTTTGCTGGCGACCACTACACGAAATTCATATGGGAGAAGTACCGCAAGCTGTCGCCGACGGCCCGACGGATGTTCGACTATTTCAGCTCGCACCGGGAGCCGTACCCGCTCAAGCTGGAAACCTTCCGCCTCATGTGCGGATCGGATTCCACCCGCGTGAAGAAGTGGCGCGAGCAGGTCGGCGAAGCCTGCGAAGAGTTGCGAGGCAGCGGCCTGGTGGAACACGCCTGGGTCAATGATGACCTGGTGCATTGCAAACGCTAGGGCCTTGTGGGGTCAGTTCCGGCTGGGGGTTCAGCAGCCAGCGCTTTACTGGCATTTCAGGAACAAGCGGGCACTGCTCGACGCACTTGCTTCGCTCAGTATCGCTCGGGACGCACGGCGCGCTCTACGAACTGCCGATAAACAGAGGATTAAAATTGACAATTGTGATTAAGGCTCAGATTCGACGGCTTGGAGCGGCCGACGTGCAGGATTTCCGCGAGATCCGATTGTCGGCCCTGAAGAAAGCTCCAGAGATGTTCGGGTCCGTTTACGAGCACGAGGAGAAAAAGCCCATGGAGGCGTTCGCTGAACGGTTGCGAGATGCCGTGGCATTCGGCGCCTACATCGACGGCGAGATCATTGGGCTGTCGGTCTTCAAACAGGAGGACGGCCCCAAGGACGCTCACAAGGCGCATCTGTCCGGCGTTTTCGTGGAGCCCGAACAGCGAGGCCGAGGGGTCGCCGGTATGCTGCTGCGGGCGTTGCCGGCGGGTTTATTGCTCGTGATGATCGTCCGACAGATTCCAACGGGAATCTGGTGGATGCGCATCTTCATCCTCGGCGCACTTAATATTTCGCTATTCTGGAGCTTGTTGTTTATTTCGGTCTACCGCCTGCCGGGCGGGGTCGCGGCGACGGTAGGCGCTGTGCAGCCGCTGATGGTCGTGTTCATCTCTGCCGCTCTGCTAGGTAGCCCGATACGATTGATGGCGGTCCTGGGGGCTATTTGCGGAACTGCGGGCGTGGCGCTGTTGGTGTTGACACCAAACGCAGCGCTAGATCCTGTCGGCGTCGCAGCGGGCCTGGCGGGGGCGGTTTCCATGGCGTTCGGAACCGTGCTGACCCGCAAGTGGCAACCTCCCGTGCCTCTGCTCACCTTTACCGCCTGGCAACTGGCGGCCGGAGGACTTCTGCTCGTTCCAGTAGCTTTAGTGTTTGATCCGCCAATCCCGATGCCTACAGGAACCAATGTTCTCGGCCTGGCGTGGCTCGGCCTGATCGGAGCGGGTTTAACCTACTTCCTTTGGTTCCGGGGGATCTC

>bbori322

CGACTCGAACCTACAGTTGTTTCCTTACTGGGCTTTCTCAGCCGGGATGGCGCTAAGAAGCTATTGCCGCCGATCTTCATATGCGGTGTGAAATACCGCACAGATGCGTAAGGAGAAAATACCGCATCAGGCGCTCTTCCGCTTCCTCGCTCACTGACTCGCTGCGCTCGGTCGTTCGGCTGCGGCGAGCGGTATCAGCTCACTCAAAGGCGGTAATACGGTTATCCACAGAATCAGGGGATAACGCAGGAAAGAACATGTGAGCAAAAGGCCAGCAAAAGGCCAGGAACCGTAAAAAGGCCGCGTTGCTGGCGTTTTTCCATAGGCTCCGCCCCCCTGACGAGCATCACAAAAATCGACGCTCAAGTCAGAGGTGGCGAAACCCGACAGGACTATAAAGATACCAGGCGTTTCCCCCTGGAAGCTCCCTCGTGCGCTCTCCTGTTCCGACCCTGCCGCTTACCGGATACCTGTCCGCCTTTCTCCCTTCGGGAAGCGTGGCGCTTTCTCAATGCTCACGCTGTAGGTATCTCAGTTCGGTGTAGGTCGTTCGCTCCAAGCTGGGCTGTGTGCACGAACCCCCCGTTCAGCCCGACCGCTGCGCCTTATCCGGTAACTATCGTCTTGAGTCCAACCCGGTAAGACACGACTTATCGCCACTGGCAGCAGCCACTGGTAACAGGATTAGCAGAGCGAGGTATGTAGGCGGTGCTACAGAGTTCTTGAAGTGGTGGCCTAACTACGGCTACACTAGAAGGACAGTATTTGGTATCTGCGCTCTGCTGAAGCCAGTTACCTTCGGAAAAAGAGTTGGTAGCTCTTGATCCGGCAAACAAACCACCGCTGGTAGCGGTGGTTTTTTTGTTTGCAAGCAGCAGATTACGCGCAGAAAAAAAGGATATCAAGAAGATCCTTTGATCTTTTCTACGGGGTCTGACGCTCAGTGGAACGAAAACTCACGTTAAGGGATTTTGGTCATGAGATTATCAAAAAGGATCTTCACCTAGATCCTTTTAAATTAAAAATGAAGTTTTAAATCAATCTAAAGTATATATGAGTAAACTTGGTCTGACAGTTACCAATGCTTAATCAGTGAGGCACCTATCTCAGCGATCTGTCTATTTCGTTCATCCATAGTTGCCTGACTCCCCGTCGTGTAGATAACTACGATACGGGAGGGCTTACCATCTGGCCCCAGTGCTGCAATGATACCGCGAGACCCACGCTCACCGGCTCCAGATTTATCAGCAATAAACCAGCCAGCCGGAAGGGCCGAGCGCAGAAGTGGTCCTGCAACTTTATCCGCCTCCATCCAGTCTATTAAACAAGTGGCAGCAACGGATTCGCAAACCTGTCACGCCTTTTGTGCCAAAAGCCGCGCCAGGTTTGCGATCCGCTGTGCCAGGCGTTAGGCGTCA

>bbspecr

TATGAAGATTTCGGTGATCCCTGAGCAGGTGGCGGAAACATTGGATGCTGAGAACCATTTCATTGTTCGTGAAGTGTTCGATGTGCACCTATCCGACCAAGGCTTTGAACTATCTACCAGAAGTGTGAGCCCCTACCGGAAGGATTACATCTCGGATGATGACTCTGATGAAGACTCTGCTTGCTATGGCGCATTCATCGACCAAGAGCTTGTCGGGAAGATTGAACTCAACTCAACATGGAACGATCTAGCCTCTATCGAACACATTGTTGTGTCGCACACGCACCGAGGCAAAGGAGTCGCGCACAGTCTCATCGAATTTGCGAAAAAGTGGGCACTAAGCAGACAGCTCCTTGGCATACGATTAGAGACACAAACGAACAATGTACCTGCCTGCAATTTGTACGCAAAATGTGGCTTTACTCTCGGCGGCATTGACCTGTTCACGTATAAAACTAGACCTCAAGTCTCGAACGAAACAGCGATGTACTGGTACTGGTTCTCGGGAGCACAGGATGACGCCTAACAATTCATTCAAGCCGACACCGCTTCGCGGCGCGGCTTAATTCAGGAGTTAAACATCATGAGGGAAGCGGTGATCGCCGAAGTATCGACTCAACTATCAGAGGTAGTTGGCGTCATCGAGCGCCATCTCGAACCGACGTTGCTGGCCGTACATTTGTACGGCTCCGCAGTGGATGGCGGCCTGAAGCCACACAGTGATATTGATTTGCTGGTTACGGTGACCGTAAGGCTTGATGAAACAACGCGGCGAGCTTTGATCAACGACCTTTTGGAAACTTCGGCTTCCCCTGGAGAGAGCGAGATTCTCCGCGCTGTAGAAGTCACCATTGTTGTGCACGACGACATCATTCCGTGGCGTTATCCAGCTAAGCGCGAACTGCAATTTGGAGAATGGCAGCGCAATGACATTCTTGCAGGTATCTTCGAGCCAGCCACGATCGACATTGATCTGGCTATCTTGCTGACAAAAGCAAGAGAACATAGCGTTGCCTTGGTAGGTCCAGCGGCGGAGGAACTCTTTGATCCGGTTCCTGAACAGGATCTATTTGAGGCGCTAAATGAAACCTTAACGCTATGGAACTCGCCGCCCGACTGGGCTGGCGATGAGCGAAATGTAGTGCTTACGTTGTCCCGCATTTGGTACAGCGCAGTAACCGGCAAAATCGCGCCGAAGGATGTCGCTGCCGACTGGGCAATGGAGCGCCTGCCGGCCCAGTATCAGCCCGTCATACTTGAAGCTAGGCAGGCTTATCTTGGACAAGAAGATCGCTTGGCCTCGCGCGCAGATCAGTTGGAAGAATTTGTTCACTACGTGAAAGGCGAGATCACCAAGGTAGTCGGCAAATAATGTCTAACAATTCGTTCAAGCCGACGCCGCTTCGCGGCGCGGCTTAACTCAAGCGTTAGAGAGCTGGGGAAGACTATGCGCGATCTGTTGAAGGTGGTTCTAAGCCTCGTACTTGCGATGGCATCGGGGCAGGCACTTGCTGACCTGCCAATTGTTTTAGTGGATGAAGCTCGTCTTCCCTATGACTACTCCCCATCCAACTACGACATTTCTCCAAGCAACTACGACAACTCCATAAGCAATTACGACAATAGTCCATCAAATTACGACAACTCTGAGAGCAACTAC

>bborirk22

GGCGCGGCGTCTATGGCGGCAAAGATGGGAGCTTCTGCGGGGCATTGGTCGTCATAAATGGCCAATTTTCGCTTGCCCTGACAGATAACGGCCTGAAGATCATGTATCTAAGCAACTAGCCTGCTCTCTAATAAAATGTTAGGAGCTTGGCTGCCATTTTTGGGGTGAGGCCGTTCGCGGCCGAGGGGCGCAGCCCCTGGGGGGATGGGAGGCCCGCGTTAGCGGGCCGGGAGGGTTCGAGAAGGGGGGGCACCCCCCTTCGGCGTGCGCGGTCACGCGCCAGGGCGCAGCCCTGGTTAAAAACAAGGTTTATAAATATTGGTTTAAAAGCAGGTTAAAAGACAGGTTAGCGGTGGCCGAAAAACGGGCGGAAACCCTTGCAAATGCTGGATTTTCTGCCTGTGGACAGCCCCTCAAATGTCAATAGGTGCGCCCCTCATCTGTCAGCACTCTGCCCCTCAAGTGTCAAGGATCGCGCCCCTCATCTGTCAGTAGTCGCGCCCCT
